# Medical Ethics in Nephrology: A Jewish Perspective

**DOI:** 10.5041/RMMJ.10241

**Published:** 2016-04-19

**Authors:** Allon N. Friedman

**Affiliations:** Department of Medicine, Indiana University School of Medicine, Indianapolis, IN, USA

**Keywords:** Bioethics, Jewish, medical ethics, nephrology, Torah

## Abstract

Jewish medical ethics is arguably the oldest recorded system of bioethics still in use. It should be of interest to practicing nephrologists because of its influence on the ethical systems of Christianity, Islam, and Western secular society; because of the extensive written documentation of rabbinical response in addressing a broad range of bioethical dilemmas; and in understanding the values of patients who choose to adhere to religious Jewish law. The goal of this review is to provide a brief overview of the basic principles underlying mainstream traditional Jewish medical ethics, apply them to common clinical scenarios experienced in nephrology practice, and contrast them with that of secular medical ethics.

## INTRODUCTION

The practice of nephrology is replete with scenarios and challenges that require the application of medical ethics. Common examples include the initiation or withdrawal of life-sustaining renal replacement therapy, balancing prolongation of life with patient suffering, and apportioning scarce resources like dialysis machines or kidney allografts.

Secular medical ethics is the most commonly applied bioethical system in the US and the Western world, but it is in fact only decades old.^1^ In contrast, the corpus of Jewish medical ethics “… constitutes a continuum of recorded deliberations and decisions dating back several millennia.”^2^ Jewish medical ethics may be of interest to nephrologists for several reasons. First, it derives from Judaism, which as the oldest monotheistic religion has influenced the ethical perspectives of Christianity, Islam, and, more broadly, Western civilization. Second, as arguably the oldest recorded bioethical system still in use, Jewish medical ethics offers a uniquely important resource in evaluating bioethical dilemmas. Third, a better understanding of Jewish bioethical approaches will help nephrologists care for patients who adhere to Halacha—the collective body of Jewish religious laws—in their daily lives.

The primary goal of this article is to describe some of the basic principles underlying Jewish medical ethics, apply them to common scenarios encountered in nephrology practice including the initiation and withdrawal of dialysis, and contrast the Jewish perspective with that of secular medical ethics. Of note, the article is meant to familiarize readers with this topic and should in no way be considered comprehensive. Rabbinical experts should be consulted for advice when evaluating those specific cases in which authoritative Halachic guidance is requested or sought. The perspective to be presented represents mainstream traditional Judaism as practiced for more than two millennia and as currently represented by Orthodox Judaism. More recent offshoots like Reform or Conservative Judaism may differ on certain points, to which the reader is referred to relevant sources.^3^

## SOURCES OF JEWISH MEDICAL ETHICS

Jewish medical ethics are derived from two foundational sources. The first is the Torah (i.e. Pentateuch or Five Books of Moses), which Jews believe was divinely revealed by God to Moses and the Jewish people at Mount Sinai over three thousand years ago. The Torah is the central text of Judaism and is known as the Written Law. It includes 613 commandments which religiously observant Jews believe are absolutely binding. The second source is the Talmud, also known as the Oral Law. It includes interpretation of the written law using logical reasoning and rabbinic insights and teachings over many centuries. It is this enormous corpus of literature and associated works spanning millennia that inform Jewish bioethics.

## DISTINGUISHING PRINCIPLES

Jewish medical ethics distinguishes itself from secular bioethics by, among other aspects, fundamental principles that are considered to be ultimately grounded in divine provenance.^4^,^5^ In addition, in contrast to Western secular culture, which emphasizes the rights of individuals, Judaism stresses individual obligations and responsibilities. Jewish ethics spurns absolutism and encourages a golden mean whenever possible. Judaism considers the value of life to be of paramount importance, preceding almost all other values. This means that patient autonomy, while important, can in specific instances be superseded by other principles.

## CASE SCENARIOS

### The Jewish Ethical Imperative to Treat the Sick

A 24-year-old healthy female presents with oliguric acute kidney injury in the setting of septic shock from pyelonephritis. She has no significant past medical history and works full-time as a bank teller. She is found to be extracellularly volume-expanded with pulmonary edema. Her serum sodium is 123 mEq/L, potassium 7.3 mEq/L, chloride 89 mEq/L, and serum bicarbonate 16 mEq/L. She does not respond to intravenous diuretic therapy and requires the initiation of renal replacement therapy for life-threatening metabolic and electrolyte derangements. Do her physicians have an ethical obligation to treat her? If so, where is this obligation derived from?

In secular medical ethics there is an implicit obligation for physicians to treat the sick. Centuries ago the Hippocratic Oath described how the physician will “use treatment to help the sick …” but “… never use it to injure or wrong them.”^6^ A more modern version of this oath used by many medical schools today states “I will apply, for the benefit of the sick, all measures that are required …”^6^

Jewish bioethics also obligates physicians to treat the sick and provides a rationale by which to understand this obligation. The first logical step in demonstrating that a nephrologist (or any physician, for that matter) has an obligation to treat a sick person is first to determine whether they even have permission to treat the patient. After all, while many illnesses arise due to a patient’s destructive behavior or habits, others appear seemingly at random. Perhaps, it could be argued, the latter illnesses are divinely ordained and should not be interfered with. That physicians do have permission is based on Talmudic commentary^7^ on a biblical verse (Exodus 21:18–19) stating that if one person injures another they are obligated to pay any financial damages incurred. Included in this responsibility is the need to pay for medical care, suggesting that medical treatment can and should be provided. Unlike in secular medical ethics permission to treat is not taken for granted but required. This is because, as noted by the outstanding Torah commentator Rashi (acronym for Rabbi Shlomo Yitzchaki, 1040–1105), some may say it was God’s will that the person became ill so therefore no one should interfere with that heavenly decree. Thus, in Jewish medical ethics there is no contradiction between providing medical treatment and God’s plan.

Since Jewish ethics permits a physician to treat a patient, the next step is to determine whether there is an obligation to do so. Judaism offers several lines of support for this concept. A highly specific one involves the *a fortiori* reasoning of the famous medieval scholar and physician Maimonides (1135–1204), used while commenting on a verse in the Torah (Deuteronomy 22:1–3). The verse states that someone finding a lost object has an obligation to return it to its original owner. Maimonides concludes that if the Torah commands us to return a lost material object to its owner, surely physicians have an obligation to restore someone’s health—something of infinitely more value—back to them. Of note, the obligation here is not simply restricted to saving someone’s life but also includes, if possible, the broader goal of restoring someone’s health. This argument is supported by another Torah verse (Leviticus 19:16) that states “one should not stand idly by the blood of one’s neighbor/friend.” An additional and more general principle supporting the obligation to provide medical care is derived from the Torah verse (Leviticus 19:18) “Love thy neighbor.”

In summary, we can confirm that under Jewish law a physician is not simply permitted to treat the ill but is obligated to do so, and not just to save a life but to restore health as well. This is in stark contrast to secular ethics as reflected in, for example, the Good Samaritan law, which protects a rescuer who voluntarily helps a victim in distress from being sued for wrongdoing. Secular ethics does not obligate all individuals to try and help a victim, as would be required under Jewish bioethics.

### The Perspective on Refusing Medical Treatment

The medical team attempts to obtain informed consent on the patient described above in order to initiate urgent renal replacement therapy. The patient, however, refuses while making it clear she understands that this may lead to her imminent death. A psychiatric evaluation finds the patient to be competent to make medical decisions. Does the patient have a right to refuse life-saving therapy?

Secular medical ethics is based on four major concepts: autonomy, beneficence, non-maleficence, and justice. In theory, none necessarily has preference over the others, while in practice the value of autonomy has assumed the dominant role. Under secular medical ethics as long as the patient has been clearly informed of the implications of refusing therapy and of alternative treatment options, and is of sound mind, she has the right to refuse renal replacement therapy even if this decision leads to a preventable death. The value of autonomy would hold true even if the patient’s decision seems irrational or self-destructive.

Using the Jewish perspective, the patient’s right to autonomy and the refusal of care can be overridden by other principles. One is the supreme value and gift of life. The Jewish view is that because God bestowed life upon the patient, whether or not she wanted it, the gift of life (unlike her material possessions) is not hers to give away. Maimonides interprets one biblical source that demonstrates this concept (Deuteronomy 4:15, “You shall safeguard your life ...”) as supporting the idea that each individual is obligated to remove any obstacle that could pose a danger to life. This verse also obligates a sick person to seek medical care. Additional support comes from a biblical verse requiring Jews to live by Torah commandments and laws (Leviticus 18:5) and, as the Talmud adds, “… not die by them.” Using *a fortiori* reasoning, the Talmud argues that if one is prohibited from sacrificing one’s life even to fulfill Torah commandments (with very rare exceptions), then surely one is prohibited from giving up one’s life when *not* fulfilling those same commandments. Finally, Maimonides also points out^8^ that maintaining one’s health is vital because God’s commandment for us to know Him and follow His edicts may be compromised when one is ill.

### The Obligation to Prolong Life

An 83-year-old man with severe inoperable coronary artery disease, congestive heart failure, pulmonary hypertension, and very advanced chronic kidney disease presents with an acute myocardial infarction that is complicated by respiratory and kidney failure. He requires the urgent initiation of renal replacement therapy for life-threatening fluid and electrolyte derangements. However, his physicians do not expect him to survive this hospitalization. Should he be initiated on dialysis if it is expected that he is unlikely to survive more than a few days?

Under secular medical ethics, the patient and/or his family can refuse dialysis treatment on the grounds that treatment is “futile,” since the medical consensus is that he is unlikely to survive much longer regardless of whether dialysis is started. In contrast, a mainstream Jewish perspective would support the initiation of renal replacement therapy in this patient. The basis for this lies in part in the general Talmudic concept of *Chayei Sha’ah*, which is defined as the short-term prolongation of life. Because in Judaism every moment of life is considered to be a divine gift of infinite value, one cannot argue that extending life only by two weeks, two days, or even two minutes is anything but meaningful. Moreover, it is understood by Torah scholars that predicting the life expectancy of patients is uncertain and prone to error. Further support arises in a Talmudic discussion^9^ as to whether one is allowed to try and save survivors in a building that has just collapsed even if this behavior desecrates the Sabbath. Observing the Sabbath is considered to be of the greatest importance to religious Jews, so breaking its rules is an issue of utmost severity. The Talmud concludes that one is allowed to violate Sabbath prohibitions even if there are doubts about whether anyone is actually trapped under the rubble. Renowned Torah scholar Rabbi Israel Meir Kagan, also known as the Chafetz Chaim (1839–1933), described how one is actually obligated to violate the Sabbath to save a life even if the person saved will be unable to do anything meaningful during the extra few moments he has been given to live. Therefore, according to Jewish bioethics even the theoretical possibility of extending a life, if even by the briefest of moments, is of the utmost importance and should be attempted. However, as described below, this value must be balanced with concerns about patient suffering.

### Balancing Preservation of Life against Patient Suffering

A 48-year-old man with metastatic pancreatic cancer is admitted to the hospital for colonic obstruction from metastatic disease. He is in unrelenting excruciating pain that is only relieved with intravenous narcotic analgesics. His disease is considered inoperable, and his life expectancy, by all accounts, is believed to be at most a matter of days. He develops acute kidney injury and requires the initiation of life-saving renal replacement therapy. Should he be initiated on dialysis?

Based on a secular perspective of medical ethics, the patient’s physicians decide not to initiate the patient on dialysis because they consider it an exercise in futility that will only prolong patient suffering. From the Jewish perspective, there is an enormous and complex body of literature that deals with the moral and legal aspects of caring for the terminally ill patient. While a range of rabbinical opinion exists on the withholding of life-sustaining treatment in such cases, the unanimous view is that the alleviation of pain and suffering should be of the highest priority. In 1995 four of the most authoritative rabbinical authorities in Israel decided that while a terminally ill patient must continue to be treated with routine supportive care (e.g. antibiotics, fluids, food, insulin, analgesics), in certain circumstances life-prolonging interventions such as dialysis could be withheld.^2^ These circumstances would involve a patient dying of a known chronic, incurable, and fatal illness in whom the intervention, which must not yet have been started, would only prolong suffering. Only suffering, and not any other factors that are sometimes accepted as indicators of “poor” quality of life (i.e. physical or mental handicaps), can be considered when determining whether to avoid prolonging the life of a terminally ill patient. Based on the rabbinical ruling mentioned above, it would therefore seem that withholding of dialysis therapy in this particular case is permissible.

### Managing Scarce Medical Resources

You are called by the dialysis nurse about an urgent dilemma. Multiple persons were admitted to the emergency department (ED) with severe crush injuries after a bus accident. One of them, an 18-year-old otherwise healthy teenager, is having massive and ongoing release of potassium with resultant life-threatening hyperkalemia and associated ventricular arrhythmias despite standard therapy. Unfortunately, the hospital’s only functioning dialysis machine is currently being used to treat severe hyperkalemia in another crush injury patient, this one an 87-year-old man with inoperable coronary artery disease, valvular disease, and other serious comorbidities. His life expectancy is believed to be less than one year. Are you permitted to take him off of dialysis early, potentially resulting in his death from hyperkalemia, in order to save the teenager’s life?

Secular medical ethics includes a branch called utilitarian ethics, which recommends directing medical resources where they will have the most long-term effect for good. Utilitarian ethics is commonly used to assess the value (e.g. quality of life years (QALYs)) of a particular treatment in health care policy or planning initiatives. Utilitarian ethics would support transferring dialysis treatment to the younger healthier patient because, though it would imperil the elder patient’s life, it would save the young woman and in doing so offer a much greater “net” quality and duration of life.

In contrast, Jewish medical ethics forbids taking the elderly patient off the dialysis machine based on at least two Talmudic principles. The first principle originates from the statement “you are not allowed to push away one life for another,”^10^ meaning one life cannot be sacrificed to save another. The second, well known, principle that could be applied to this scenario is derived from the concept of possession as used to resolve monetary disputes.^11^ As in the secular legal world where “possession is nine-tenths of the law,” the concept of possession also holds great sway in Judaism. Since the elderly patient currently possesses usage of the dialysis machine, withdrawing the machine and therapy against his will would violate this basic tenet.

## SUMMARY

Jewish medical ethics is an ancient system of belief that is applicable to contemporary bioethical dilemmas in all fields of medicine, including nephrology. It distinguishes itself from secular medical ethics in numerous ways, including by being grounded in divine provenance and the central holy texts and values of the Jewish people. It obligates patients to seek medical care and physicians to provide it. It holds that life is a divine gift of supreme importance. While the prolongation of life is therefore a central tenet of Jewish bioethics, this principle can be balanced against patient suffering. Finally, in terms of managing scarce resources, Jewish bioethics rejects the concept of utilitarian ethics.
